# Gabapentin and intrathecal morphine combination therapy results in decreased oral narcotic use and more consistent pain scores after posterior spinal fusion for adolescent idiopathic scoliosis

**DOI:** 10.1186/s13018-021-02525-z

**Published:** 2021-11-15

**Authors:** Ying Li, Jennylee Swallow, Christopher Robbins, Michelle S. Caird, Aleda Leis, Rebecca A. Hong

**Affiliations:** 1 Department of Orthopaedic Surgery, C.S. Mott Children’s Hospital, Michigan Medicine, Ann Arbor, MI USA; 2 Department of Surgery, Indiana University School of Medicine, Indianapolis, IN USA; 3 Department of Epidemiology, University of Michigan, Ann Arbor, MI USA; 4 Department of Anesthesiology, C.S. Mott Children’s Hospital, Michigan Medicine, 1540 E. Hospital Drive, SPC 4245, Ann Arbor, MI 48109-4245 USA

**Keywords:** Adolescent, Scoliosis, Opioids, Gabapentin, Pain, PONV (postoperative nausea and vomiting)

## Abstract

**Background:**

Gabapentin and intravenous patient-controlled analgesia (PCA) can reduce postoperative pain scores, postoperative opioid use, and time to completing physical therapy compared to PCA alone after posterior spinal fusion (PSF) for adolescent idiopathic scoliosis (AIS). Gabapentin combined with intrathecal morphine has not been studied. The primary purpose of this retrospective study was to evaluate whether perioperative gabapentin and intrathecal morphine provide more effective pain control than intrathecal morphine alone after PSF for AIS.

**Methods:**

Patients aged 11 to 18 years who underwent PSF for AIS were identified. Patients who received intrathecal morphine only (ITM group) were matched by age and sex to patients who received intrathecal morphine and perioperative gabapentin (ITM+GABA group). The ITM+GABA group received gabapentin preoperatively and for up to 2 days postoperatively. Both groups received oxycodone and the same non-narcotic adjuvant medications.

**Results:**

Our final study group consisted of 50 patients (25 ITM, 25 ITM+GABA). The ITM+GABA group had significantly lower mean total oxycodone consumption during the hospitalization (0.798 vs 1.036 mg/kg, *P*<0.015). While the ITM group had a lower mean pain score between midnight and 8 am on POD 1 (2.4 vs 3.7, *P*=0.026), pain scores were significantly more consistent throughout the postoperative period in ITM+GABA group. The ITM+GABA group experienced less nausea/vomiting (52% vs 84%, *P*=0.032) and pruritus (44% vs 72%, *P*=0.045). Time to physical therapy discharge and length of hospital stay were similar.

**Conclusion:**

Addition of gabapentin resulted in reduced oral opioid consumption and more consistent postoperative pain scores after PSF for AIS. The patients who received intrathecal morphine and gabapentin also experienced a lower rate of nausea/vomiting and pruritus.

**Trial registration:**

All data was collected retrospectively from chart review, with institutional IRB approval. Trial registration is not applicable.

## Background

Multimodal pain management has been found to improve the quality of analgesia and reduce medication-related side effects after posterior spinal fusion (PSF) for adolescent idiopathic scoliosis (AIS) [1–7]. Prior studies have shown that perioperative gabapentin, an effective drug for neuropathic pain, combined with intravenous patient-controlled analgesia (PCA) reduces initial postoperative pain scores, postoperative opioid use, and time to completing physical therapy goals compared to PCA alone after PSF for AIS [1, 3–5]. Postoperative pain is not a Food and Drug Administration-approved indication for gabapentin usage. However, off-label use of gabapentin for postoperative pain in adults and children is endorsed as a “strong recommendation, moderate-quality evidence” by the American Society of Anesthesiologists, American Society of Regional Anesthesia and Pain Medicine, and American Pain Society [8].

Intrathecal morphine has been found to provide satisfactory pain control after PSF for AIS [9–15]. However, the patients in the majority of these studies also received a PCA [9, 11, 13], continuous intravenous morphine infusion [15], or continuous epidural infusion [14] for pain control postoperatively. We previously demonstrated that intrathecal morphine combined with oral analgesics provides safe and effective pain control after PSF for AIS [10, 12]. Similar to other reports on intrathecal morphine use in this patient population [11, 13, 15], minor adverse events, such as postoperative nausea/vomiting and pruritus, occurred in ≥ 2/3 of our patients.

In 2018, our institution initiated oral gabapentin administration in the perioperative period with the intention of improving pain control while reducing opioid-related side effects. The primary purpose of this retrospective study was to evaluate whether the previously unstudied combination of perioperative gabapentin and intrathecal morphine provides more effective pain control than intrathecal morphine alone after PSF for AIS. The secondary purpose was to compare time to physical therapy discharge, length of hospital stay, and adverse events. We hypothesized that addition of gabapentin would result in effective pain control, reduced oral opioid consumption, and decreased opioid-related side effects.

## Methods

Approval from our institutional review board was obtained (HUM00094642) prior to retrospective data collection. Our historical controls were patients we had previously identified, aged 11 to 18 years, who had undergone PSF for AIS between February 2017 and September 2018. These patients received intrathecal morphine alone (ITM group) and were matched by age ± 2 years and sex to patients who received intrathecal morphine and perioperative gabapentin (ITM+GABA group) during the period of February 2018 through September 2018. The patients were not paired. None of these patients were included in our previous study. Inclusion criteria were patients with AIS who had undergone primary PSF and received intrathecal morphine with or without perioperative gabapentin. Exclusion criteria were patients with non-idiopathic scoliosis, American Society of Anesthesiologists (ASA) physical status 4, and previous spinal fusion.

Our intrathecal morphine for PSF for AIS institutional standard of care protocol has been previously published [12]. Briefly, patients underwent intrathecal morphine injection by the anesthesiologist after induction of anesthesia and before incision. Due to concerns for potential oversedation with the addition of gabapentin, the 2018 protocol provides for administration of a lower dose of intrathecal morphine in the ITM+GABA group. Postoperatively, all patients were scheduled to receive 0.1 mg/kg of oral oxycodone (maximum 5 mg) 16 h after the injection of intrathecal morphine and no intravenous narcotics were ordered for the postoperative period. Both groups received the same non-narcotic adjuvant pain medications (acetaminophen, ketorolac, diazepam) other than gabapentin per standard of care. Oral opioids and adjuvant medications were managed by the pediatric anesthesia acute pain service. Pain was assessed using a self-reported numeric rating scale (range 0–10, where 0 is no pain and 10 is the worst pain imaginable) and sedation was evaluated using the University of Michigan Sedation Scale [16] (range 0–4, where 4 is unarousable). Oversedation was defined as recorded UMSS ≥ 2 or qualitative statements indicating a similar level of sedation in acute pain service notes.

Electronic medical records were reviewed retrospectively by a trained research assistant to collect patient demographics; surgical data; pain and sedation scores; administration of analgesics, antiemetics, and antipruritics; time to Foley catheter removal, ambulation, and physical therapy discharge; length of hospital stay; and adverse events. Pain scores were recorded for the following time periods: time in post-anesthesia care unit (PACU), PACU discharge to midnight, postoperative day (POD) 1 midnight to 08:00, POD 1 0:801 to 16:00, POD 1 16:01 to midnight, and POD 2.

Statistical analysis was conducted using SPSS v. 25.0 (IBM Corp, Armonk, NY). The demographic, clinical, and related characteristics across patients were described by using raw counts, percentages, measures of central tendency (e.g., mean, median or mode), and measures of data dispersion (e.g., 95% CIs) where appropriate. Primary analyses for between-group comparisons for pain or time variables consisted of unadjusted independent samples t-test or Mann-Whitney U test for non-parametric data. Tests of within-group differences for pain or time variables were conducted using paired t-tests or the Wilcoxon signed-rank test for non-parametric data. Chi-square analysis was used to test associations between groups and categorical variables such as adverse events coded yes/no. All tests were conducted two-tailed with statistical significance set at *P* < 0.05 (95% confidence interval).

## Results

Our final study group consisted of 50 patients (25 ITM group and 25 ITM+GABA group). Demographic and surgical data were similar between the groups (Table 1). The ITM and ITM+GABA groups received an average dose of 7.8 μg/kg (range 6.0–8.6 μg/kg, maximum 600 μg) and 5.7 μg/kg of intrathecal morphine (range 2.5–8.0 μg/kg, maximum 530 μg), respectively. The ITM+GABA group received a mean dose of 7.8 mg/kg of gabapentin (range 4.0–11.5 mg/kg, maximum 700 mg) within 1 h before going to the operating room, followed by a mean of 5.1 mg/kg/day of gabapentin in divided doses (range 2.4–15.3 mg/kg/day, maximum 1050 mg/day) for up to 2 days postoperatively starting on POD 1. Both groups received similar intravenous and oral analgesics intraoperatively and in the PACU (Table 2). All patients were transferred to the PACU after surgery and were then admitted to the general care floor. No patients were admitted to the intensive care unit.

**Table 1:** Demographic and surgical data

	ITM + gabapentin (***n*** = 25)	ITM only (***n*** = 25)
Age (years)	14.8 ± 1.9	14.8 ± 1.8
Male [n (%)]	5 (20%)	5 (20%)
Weight (kg)	62.0 ± 19.6	54.8 ± 10.7
ASA Classification [n (%)]		
1	8 (32%)	6 (24%)
2	15 (60%)	19 (76%)
3	2 (8%)	0 (%)
Mean number of levels fused	9 (range 4–12)	10 (range 5–13)
Estimated blood loss (mL)	287 ± 261	336 ± 294

Data are presented as mean ± standard deviation, unless otherwise stated

*ITM* intrathecal morphine, *ASA* American Society of Anesthesiologists

**Table 2:** Patients in each group who received intravenous or oral analgesics intraoperatively and in the PACU

	Intraoperatively		***P***	PACU		***P***
	ITM + gabapentin	ITM only		ITM + gabapentin	ITM only	
Remifentanil	20 (80%)	18 (72%)	0.508	N/A	N/A	
Dexmedetomidine	5 (20%)	10 (40%)	0.123	N/A	N/A	
Fentanyl	21 (84%)	22 (88%)	1.000	4 (16%)	5 (20%)	1.000
Acetaminophen	18 (72%)	22 (88%)	0.289	2 (8%)	1 (4%)	1.000
Ketorolac	22 (88%)	22 (88%)	1.000	1 (4%)	2 (8%)	1.000
Diazepam^a^	1 (4%)	3 (12%)	0.609	9 (36%)	11 (44%)	0.564
Ketamine	11 (44%)	11 (44%)	1.000	0	0	
Midazolam	20 (80%)	20 (80%)	1.000	0	0	

Values are shown as n (%)

*ITM* intrathecal morphine, *PACU* post-anesthesia care unit

^a^Intravenous diazepam was on shortage for most of the study time period. The majority of these doses were oral (3/4 administered intraoperatively and 17/20 administered in PACU)

Mean postoperative intravenous and oral analgesic doses administered after PACU discharge are presented in Table 3. The ITM+GABA patients had significantly lower mean oxycodone usage on POD 2 (0.331 vs 0.471 mg/kg/day, *P* = 0.047) and significantly lower mean total oxycodone consumption throughout the entire hospitalization (0.798 vs 1.036 mg/kg, t = 2.54, *P* < 0.015, CI − 0.427, − 0.048).

**Table 3:** Mean postoperative intravenous and oral analgesic doses (mg/kg) administered in each group

	POD 0			POD 1			POD 2		
	ITM + gabapentin	ITM only	***P***	ITM + gabapentin	ITM only	***P***	ITM + gabapentin	ITM only	***P***
Oxycodone	0.003 ± 0.016	0.013 ± 0.037	0.274	0.464 ± 0.212	0.552 ± 0.117	0.222	0.331 ± 0.230	0.471 ± 0.146	**0.047**
Acetaminophen	16.72 ± 7.98	15.08 ± 9.89	0.800	47.75 ± 13.70	49.38 ± 14.93	0.662	33.33 ± 18.60	40.99 ± 16.52	0.140
Ketorolac	0.336 ± 0.189	0.326 ± 0.186	0.691	1.031 ± 0.412	1.140 ± 0.279	0.200	0.475 ± 0.334	0.636 ± 0.361	0.159
Diazepam	0.023 ± 0.027	0.015 ± 0.031	0.086	0.160 ± 0.093	0.138 ± 0.097	0.438	0.128 ± 0.094	0.159 ± 0.100	0.235

Data are presented as mean ± standard deviation

*ITM* intrathecal morphine, *POD* postoperative day

**Fig. 1:**
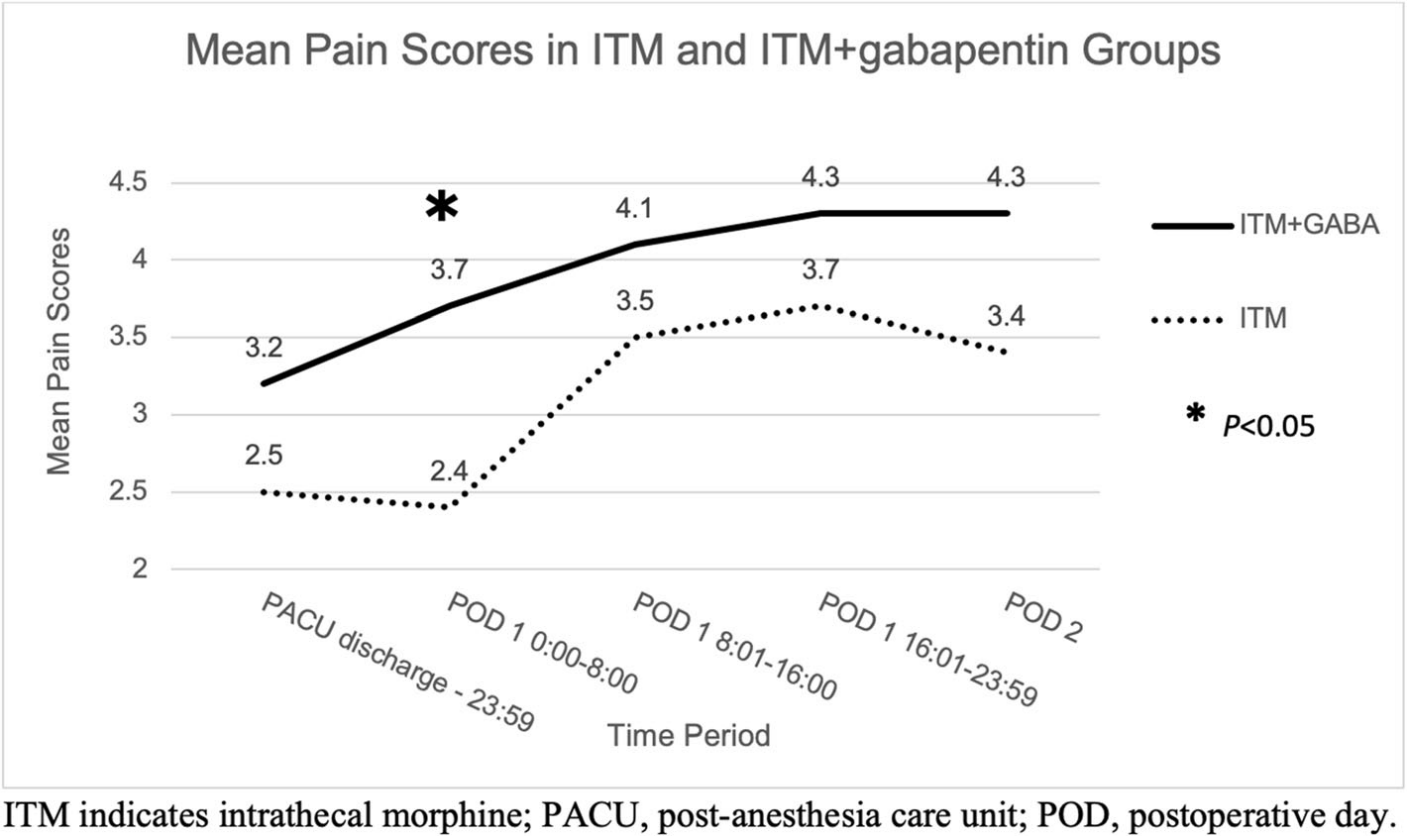
Mean pain scores in ITM and ITM+gabapentin groups. Legend: Mean pain scores for both groups. The only significant difference in mean pain scores was during POD 1 0:00–8:00. ITM, intrathecal morphine; PACU, post-anesthesia care unit; POD, postoperative day

The highest, lowest, and mean pain scores are shown in Table 4. While the ITM group had a lower mean pain score between midnight and 8 am on POD 1 (2.4 vs 3.7, t = 2.29, *P* = 0.026, CI 0.161, 2.43), pain scores appeared to be more consistent throughout the postoperative period in the ITM+GABA group (Fig. 1). Table 5 shows the difference in mean pain scores between time periods in each group. The ITM+GABA patients had similar pain scores across all time periods, whereas the ITM patients had a significant difference in pain scores when the POD 1 4 pm to midnight time period was compared to the PACU discharge to midnight time period (Δ 1.3, t = 3.82, *P* = 0.028, CI 0.59, 1.96) and the POD 1 midnight to 8 am time period (Δ 1.3, t = 3.98, *P* = 0.005, CI 0.63, 1.99).

**Table 4:** Highest, lowest, and mean reported numeric rating scale pain scores (0–10) for both groups for the first 2 postoperative days

	Highest pain score			Lowest pain score			Difference between highest and lowest pain scores			Mean pain score		
	ITM + gabapentin	ITM only	***P***	ITM + gabapentin	ITM only	***P***	ITM + gabapentin	ITM only	***P***	ITM + gabapentin	ITM only	***P***
PACU discharge–23:59	4.6 ± 2.9	3.9 ± 2.5	0.409	2.0 ± 1.8	1.2 ± 1.5	0.095	2.6 ± 2.1	2.7 ± 2.1	0.765	3.2 ± 2.1	2.5 ± 1.8	0.208
POD 1												
00:00–08:00	5.0 ± 2.4	3.5 ± 2.5	**0.035**	2.6 ± 2.0	1.7 ± 1.9	0.292	2.4 ± 2.2	1.8 ± 1.3	0.256	3.7 ± 1.9	2.4 ± 2.1	**0.026**
08:01–16:00	5.6 ± 2.4	5.4 ± 1.8	0.740	2.3 ± 1.5	2.1 ± 1.4	0.119	3.3 ± 2.3	3.3 ± 1.9	0.865	4.1 ± 1.7	3.5 ± 1.5	0.201
16:01–23:59	6.0 ± 2.4	5.4 ± 2.5	0.412	2.9 ± 1.6	2.0 ± 1.2	0.056	3.1 ± 2.5	3.4 ± 2.4	0.701	4.3 ± 1.6	3.7 ± 1.6	0.198
POD 2	6.7 ± 2.2	6.1 ± 2.5	0.462	2.0 ± 1.6	1.2 ± 1.4	**0.012**	4.7 ± 2.1	5.0 ± 2.5	0.175	4.3 ± 1.6	3.4 ± 1.6	0.051

Data are presented as the mean ± standard deviation

*ITM* intrathecal morphine, *PACU* post-anesthesia care unit, *POD* postoperative day

**Table 5:** Difference in mean pain scores between time periods in each group for the first 2 postoperative days

	ITM+gabapentin				ITM only			
	POD 1			POD 2	POD 1			POD 2
	00:00–08:00	08:01–16:00	16:01–23:59		00:00–08:00	08:01-16:00	16:01–23:59	
PACU discharge–23:59	0.5	0.9	1.1	1.1	0	1.0	1.3*	0.9
POD 1 00:00–08:00		0.4	0.6	0.5		1.1	1.3**	0.9
08:01–16:00			0.3	0.2			0.3	0.1
16:01–23:59				0.1				0.4

Values shown as difference in pain scores between two time periods

*ITM*, intrathecal morphine; *PACU*, post-anesthesia care unit; *POD*, postoperative day

*p < 0.05

**p < 0.01

There was no difference between the ITM and ITM+GABA groups in time to Foley catheter removal (19.3 ± 5.5 vs 17.5 ± 2.5 h, *P*=0.139), ambulation (20.7 ± 8.1 vs 19.3 ± 5.5 h, *P*=0.475), physical therapy discharge (2.3 ± 0.5 vs 2.0 ± 0.7 days, *P*=0.172), and length of hospital stay (2.6 ± 0.6 vs 2.4 ± 0.7 days, *P*=0.387).

The ITM+GABA patients experienced a significantly lower rate of adverse events. The ITM+GABA group had less postoperative nausea/vomiting (13 [52%] vs 21 [84%], χ^2^ *=* 5.88, *P* = 0.015) and pruritus (11 [44%] vs 18 [72%], χ^2^ *=* 4.02, *P*=0.045). Respiratory depression managed with nasal cannula oxygen (3 [12%] vs 6 [24%], *P*=0.269) and oversedation (4 [16%] vs 5 [20%], *P*=0.295) were similar between the ITM and ITM+GABA groups. No patients experienced oversedation to the point of escalation of care status or transfer to the intensive care unit, and no patients received naloxone or flumazenil in the postoperative period.

## Discussion

To our knowledge, this retrospective study is the first to evaluate the effectiveness of perioperative gabapentin combined with intrathecal morphine for pain control after PSF for AIS. We previously showed that intrathecal morphine combined with oral analgesics provides safe and effective pain control after PSF for AIS [10, 12]. When our institution initiated our intrathecal morphine for PSF for AIS protocol in 2014, anesthesiologists administered 12 μg/kg of intrathecal morphine (maximum 1000 μg) [10]. Ninety percent of patients experienced postoperative nausea/vomiting and 40% of patients had pruritus. The protocol was modified by decreasing the dose of intrathecal morphine to 8–10 μg/kg (maximum 800 μg) and implementing more consistent dosing of adjuvant medications (acetaminophen, ketorolac, diazepam) [12]. While patients had lower and more consistent pain scores after implementation of the updated protocol, nausea/vomiting and pruritus were still experienced by 89% and 64% of patients, respectively. Although the rates of postoperative nausea/vomiting and pruritus after decreasing the dose of intrathecal morphine were similar to our historical control group who received a hydromorphone epidural infusion [10], we strove to further improve our protocol to reduce opioid-related side effects while providing effective analgesia.

In 2018, our institution initiated oral gabapentin administration in the perioperative period. Due to concerns for potential oversedation, standard of care was to decrease the dose of intrathecal morphine administered. The results of this retrospective study show that while addition of gabapentin as adjuvant therapy did not seem to have an effect on pain scores in the early postoperative period, it did result in reduced oral opioid consumption and more consistent postoperative pain scores throughout the hospitalization after PSF for AIS. This postoperative opioid-sparing effect was seen even despite patients in this group receiving a lower dose of intrathecal morphine in the operating room. The patients who received intrathecal morphine alone had significantly higher pain scores in the afternoon and evening of POD 1 compared to earlier postoperative time periods. This may be secondary to increased pain after mobilization with physical therapy. The patients who received gabapentin would have had one to two postoperative doses by the afternoon of POD 1 and this may explain the more consistent pain scores in that group.

Previous studies have assessed the effect of perioperative gabapentin combined with intravenous PCA after PSF for AIS and showed a single dose of preoperative gabapentin did not result in a difference in opioid use or pain scores compared to placebo [17]. A randomized, double-blind, controlled trial conducted by Rusy et al. demonstrated lower pain scores in PACU and the morning after surgery in patients who received a preoperative dose of gabapentin and postoperative gabapentin for 5 days [3]. Patients who received perioperative gabapentin also had reduced postoperative morphine consumption during the first two postoperative days. As such, the authors recommended continuing gabapentin for only the first 2 days after surgery. Similarly, Trzcinski et al. retrospectively found that patients who received perioperative gabapentin had improved pain scores and decreased opioid use for 48 to 72 h after surgery [5]. Thomas et al. demonstrated a decrease in time to complete physical therapy goals when patients received perioperative gabapentin but there was no difference in length of hospital stay [4].

Choudhry et al. retrospectively showed that PSF patients who received perioperative gabapentin combined with intravenous PCA had decreased total PCA doses, decreased morphine use on POD 1, and shorter time to transition to orals compared to patients who received PCA alone [1]. However, postoperative pain scores, time to ambulation, and length of hospital stay were similar. These authors found that addition of a clonidine transdermal patch for 7 days postoperatively to perioperative gabapentin and intravenous PCA resulted in the shortest time to transition to orals, shortest time to ambulation, and shortest length of hospital stay, but there was no difference in total PCA doses and morphine use compared to patients who received gabapentin and intravenous PCA.

Although we did not observe a shorter time to ambulation, physical therapy discharge, or length of hospital stay with the addition of perioperative gabapentin to intrathecal morphine, PSF patients who are managed according to our intrathecal morphine for PSF for AIS protocol appear to achieve these goals earlier than what has been reported by other authors. The mean time to ambulation for both groups in our study was 19 to 21 h, mean time to physical therapy discharge was 2.0 to 2.3 days, and mean length of hospital stay was 2.4 to 2.6 days. While Thomas et al. found a decrease in time to complete physical therapy goals when patients received perioperative gabapentin combined with intravenous PCA, only 28% of those patients ambulated on POD 1, 52% of those patients completed all physical therapy goals on POD 2, and mean length of hospital stay was 3 days [4]. Similarly, in Choudhry et al.’s study, the gabapentin and gabapentin plus clonidine groups had a mean time to ambulation of 36 h and 27 h, and a mean length of hospital stay of 87 h and 77 h, respectively [1].

Prior studies have shown no difference in the rate of adverse events, including postoperative nausea/vomiting, pruritus, and oversedation, with the addition of perioperative gabapentin to intravenous PCA after PSF for AIS [1, 3, 5]. Nausea/vomiting is a common adverse event after PSF. Trzcinski et al. reported nausea/vomiting in 75% of their entire retrospective cohort [5]. The patients who received perioperative gabapentin in our study experienced a significantly lower rate of postoperative nausea/vomiting and pruritus, possibly secondary to receiving a lower dose of intrathecal morphine. However, this difference may also be directly related to antiemetic effects of gabapentin at central nervous system sites [18] as some prior studies have shown that it appears to have antiemetic effects in at least some patient populations [19], including spinal surgery [20]. In our ITM+GABA group, 52% still either received an antiemetic medication or had documented nausea/vomiting and 44% either received antipruritic medication or had documented pruritus. It can be difficult to retrospectively determine the rate of these minor adverse events. In addition to documentation of an adverse event in the medical record, we also considered administration of an antiemetic or antipruritic as evidence of nausea/vomiting or pruritus. However, it can be difficult to know whether these medications were administered for prophylaxis or treatment so the rate of these adverse events may have been over-reported. Also, at our institution, ondansetron is ordered as treatment for both nausea/vomiting and pruritus, making it difficult to tell retrospectively for which indication it was given.

Another limitation of this study is inconsistent perioperative gabapentin dosing. Per institutional protocol, gabapentin was to be administered at 10 mg/kg preoperatively followed by 5 mg/kg three times daily for 2 days beginning on POD 1. In reality, there was a wide range of gabapentin dosing due to many factors. One was the lack of prompt availability of liquid gabapentin in our preoperative area for patients who were not able to swallow pills. Additionally, the pediatric anesthesiology group at our institution is large (>35 anesthesiologists) with variable preferences for gabapentin dosing and uncertainty about the impact that the addition of gabapentin might have on their preferred anesthetic technique for these cases. We found wide variability in gabapentin dosing in published reports on perioperative gabapentin combined with intravenous PCA in PSF patients with AIS [1, 3–5, 17]. We hope that the retrospective data we have collected will allow us to implement a standard perioperative gabapentin protocol for all patients undergoing PSF for AIS at our institution and we have since worked with our operating room pharmacy to increase the availability of liquid gabapentin in the preoperative area. Another limitation is the small sample size. Our study may have been underpowered to detect differences in some of our outcomes. Lastly, this was a retrospective study so data collection was dependent on accurate documentation in the medical record.

## Conclusions

In conclusion, while perioperative gabapentin combined with intrathecal morphine results in reduced oral opioid consumption and more consistent postoperative pain scores after PSF for AIS compared to intrathecal morphine alone, there does not seem to be an effect on pain scores in the early postoperative period. Patients who received intrathecal morphine and gabapentin did, however, experience a significantly lower rate of postoperative nausea/vomiting and pruritus. Given the current opioid epidemic, it is encouraging to see an opioid-sparing effect with the use of gabapentin following this painful surgery in adolescents. Future studies are indicated to optimize gabapentin dosing with intrathecal morphine and to continue to improve rates of postoperative nausea and vomiting.

## Data Availability

The datasets used and/or analyzed during the current study are available from the corresponding author on reasonable request. The software application and code used for the statistical analysis are available from the corresponding author on reasonable request.

## Abbreviations

PSF: Posterior spinal fusion
AIS: Adolescent idiopathic scoliosis
PCA: Patient-controlled analgesia
ITM Group: Intrathecal morphine group—patients who received intrathecal morphine for their surgery
ITM + GABA Group: Intrathecal morphine + gabapentin group—patients who received intrathecal morphine + gabapentin for their surgery
ASA: American Society of Anesthesiologists
UMSS: University of Michigan Sedation Scale
PACU: Post-anesthesia care unit
POD: Postoperative day
